# The ghrelin-GHSR-1a pathway inhibits high glucose-induced retinal angiogenesis in vitro by alleviating endoplasmic reticulum stress

**DOI:** 10.1186/s40662-022-00291-5

**Published:** 2022-06-07

**Authors:** Rong Li, Guomin Yao, Lingxiao Zhou, Min Zhang, Jin Yan

**Affiliations:** 1 Department of Ophthalmology, The First Affiliated Hospital of Xi’an Medical University, No.48 West Fenghao Road, Xi’an, 710077 Shaanxi China; 2 Department of Endocrinology, The First Affiliated Hospital of Xi’an Medical University, No.48 West Fenghao Road, Xi’an, 710077 Shaanxi China; 3 College of Medical Technology of Xi’an Medical University, No.1 Xinwang Road, Xi’an, 710021 Shaanxi China

**Keywords:** Ghrelin, GHSR-1a, Endoplasmic reticulum stress, Angiogenesis, Retinal neovascularization, Diabetic retinopathy

## Abstract

**Background:**

To investigate the effect of ghrelin, a brain-gut peptide hormone, on high glucose-induced retinal angiogenesis in vitro and explore its association with endoplasmic reticulum (ER) stress.

**Methods:**

Human retinal microvascular endothelial cells (HRMECs) were first divided into control and high-glucose groups, and the mRNA and protein expression levels of the receptor for ghrelin [growth hormone secretin receptor 1a, (GHSR-1a)] in cells were determined. HRMECs were then treated with high glucose alone or in combination with ghrelin or siGHSR-1a, and cell viability, migration, tube formation and the expression of the ER stress-related proteins PERK, ATF4 and CHOP were detected. Finally, to clarify whether the effects of ghrelin are related to ER stress, tunicamycin, an inducer of ER stress, was used to treat HRMECs, and cell viability, cell migration, and tube formation were evaluated.

**Results:**

GHSR-1a expression in HRMECs at both the mRNA and protein levels was inhibited by high-glucose treatment. Under high-glucose conditions, ghrelin promoted cell viability and inhibited migration and tube formation, which were blocked by siGHSR-1a treatment. Ghrelin inhibited the increases in the protein levels of p-PERK, ATF4 and CHOP induced by high-glucose treatment, and combination treatment with siGHSR-1a reversed this effect of ghrelin. When tunicamycin was added, the effects of ghrelin on cell viability, migration and tube formation were all weakened.

**Conclusions:**

This study experimentally revealed that ghrelin can inhibit high glucose-induced retinal angiogenesis in vitro through GHSR-1a, and alleviation of ER stress may be one of the mechanisms underlying this effect.

## Background

Diabetic retinopathy (DR) is the major eye complication of diabetes, causing irreversible blindness among working-age people worldwide [1]. Hyperglycemia and other factors lead to dysfunction of retinal microvascular endothelial cells, which eventually results in retinal neovascularization in the late stage of the disease, and to secondary retinal detachment and vitreous hemorrhage leading to vision loss [2]. Normal intercellular interactions are altered by diabetes leading to profound vascular abnormalities, loss of the blood-retinal barrier and impaired neuronal function [3]. Among the factors involved in the complex retinal pathophysiology of DR, oxidative stress, inflammation, and angiogenesis-related factors play critical roles [4]. At present, laser photocoagulation, anti-neovascularization drugs [5], anti-inflammatory drugs [6] and vitrectomy are the mainstays of DR treatment, however, a risk of damage to healthy retinal tissues exists, and the efficacy of these treatment strategies remains insufficient. Therefore, exploring the pathological mechanism and new treatment strategies for DR has become increasingly attractive in recent years [7, 8].

Ghrelin is a growth hormone-releasing peptide that regulates physiological processes associated with energy homeostasis such as appetite, insulin signaling, glucose metabolism, and adiposity [9]. It is widely distributed in human cells, and mainly binds to the growth hormone secretagogue receptor-1a (GHSR-1a), a seven transmembrane G protein-coupled receptor. Recent studies revealed that ghrelin-GHSR-1a exert an increasing number of biological effects; therefore, ghrelin is a promising therapeutic agent for many diseases [10–12]. Ghrelin can reach ocular tissues through the blood-brain barrier [13, 14]. In addition, ghrelin and its receptors are also expressed in the iris, ciliary body, retina, and other ocular tissues, showing therapeutic potential in glaucoma and retinal neovascular diseases [14, 15]. Moreover, alterations in plasma ghrelin levels and the functioning of other components of the ghrelin system have been proposed as potential contributors to diabetes and its complications, and targeting the ghrelin system has been proposed as a novel therapeutic strategy for diabetes [16]. However, there are few reports on the effects of ghrelin in DR [17]. When the ability of cells to properly fold and post-translationally modify secretory and transmembrane proteins is impeded by various insults, accumulation of misfolded proteins in the endoplasmic reticulum (ER) occurs, a condition called ER stress [18]. Emerging evidence has demonstrated clear associations between ER stress-related physiological functions and the pathogenesis of DR [19]. In addition, recent studies point to the regulatory effects of ghrelin on ER stress in many illnesses [20]. In this study, we aimed to observe the effects of ghrelin on retinal vascular endothelial cell angiogenesis as well as the association of ghrelin with ER stress under the influence of high concentrations of glucose in vitro, with the purpose of laying a foundation for further study of the role and mechanism of ghrelin in DR.

## Methods

### Cell culture and treatment

Human retinal microvascular endothelial cells (HRMECs; Shanghai Zhong Qiao Xin Zhou Biotechnology Co., Ltd., China) were cultured in M199 medium (Procell, Wuhan, China) containing 10% fetal bovine serum (FBS; Excell Bio, Shanghai, China) at 37 °C in humidified 95% room air with 5% CO_2_. To detect the expression of GHSR-1a in cells, HRMECs were randomly divided into a control group (C group; cultured in M199 medium with 5.5 mM glucose) and a high-glucose group (HG group; cultured in M199 medium with 30 mM glucose); the cells in both groups were treated for 48 h.

Then, the cells were treated with high glucose and different concentrations of ghrelin (0 nM, 2.5 nM, 5 nM, 10 nM, 20 nM, and 40 nM) (Glpbio, Montclair, CA, USA) for 12 h, 24 h, 48 h and 72 h. The optimal concentration and action time of ghrelin based on the results of the cell counting kit-8 (CCK-8) assay (10 nM, 48 h) was used in the subsequent experiments. To evaluate cell viability, migration and tube formation, HRMECs were randomly divided into five groups and incubated for 48 h: the C group, HG group, ghrelin group (cultured in M199 medium with 30 mM glucose and 10 nM ghrelin), ghrelin + siGHSR-1a group (transfected with GHSR-1a-specific siRNA for 24 h and then cultured in M199 medium with 30 mM glucose and 10 nM ghrelin) and ghrelin + NC-siGHSR-1a group (transfected with a nonspecific siRNA sequence for 24 h and then cultured in M199 medium with 30 mM glucose and 10 nM ghrelin).

Finally, tunicamycin (MedChemExpress, Newark, NJ, USA), an inducer of ER stress, was utilized in the ghrelin + tunicamycin group (cultured in M199 medium with 30 mM glucose, 10 nM ghrelin and 10 μM tunicamycin). Cell viability, migration, and tube formation of HRMECs in the C group, HG group, ghrelin group and ghrelin + tunicamycin group were compared.

### Immunofluorescence staining of GHSR-1a

HRMECs on a climbing slide were rinsed with PBS and fixed with 4% paraformaldehyde for 15 min. The slide was then treated with 0.5% Triton X-100 at room temperature for 20 min after rinsing with PBS. After washing and drying, goat serum was dripped onto the slide (Boster, Wuhan, China) at room temperature for 30 min. Then, a primary antibody against GHSR-1a (1:100, Affinity, OH, USA) was dripped onto each slide and incubated at 4 °C overnight prior to incubation with Cy3-labelled goat anti-rabbit IgG (1:100, Boster, Wuhan, China) as a secondary antibody at 37 °C for 1 h. The slide was then incubated with DAPI (Beyotime, Shanghai, China) in the dark for 5 min and sealed with an anti-fluorescence quenching sealing solution. Images were acquired under a fluorescence microscope (Olympus BX53, Japan), and three images acquired under high-power (400 ×) magnification were selected from each group and used for analysis of the mean optical density with the IPP6.0 software (Media Cybernetics, Inc., USA).

### Cell viability

The viability of HRMECs was determined by a CCK-8 assay (Elabscience, Wuhan, China) according to manufacturer’s instructions. In brief, cells were seeded in each well of a 96-well plate at a density of 5 × 10^3^ cells/mL. Then, after different treatments for 48 h, 10 μL of CCK-8 reagent was added to each well. After incubation at 37 °C for 4 h, the absorbance value (A) of each well at 450 nm was estimated in a microplate reader. Cell viability (%) was calculated as (A_experiment group_ − A_blank group_)/(A_control group_ − A_blank group_) × 100, where A_experiment group_ is the absorbance value of treated cells, A_blank group_ is the absorbance value of cell-free medium, and A_control group_ is the absorbance value of untreated cells.

### siRNA transfection

Cells in the logarithmic growth phase were seeded in 6-well plates and placed in an incubator at 37 °C for 24 h. At approximately 50% confluence, HRMECs were transfected with a GHSR-1a-specific siRNA (20 μM; Tsingke Biotechnology Co., Ltd., Beijing, China) using Lipofectamine™ 2000 Reagent (Invitrogen, Carlsbad, CA, USA) in accordance with the manufacturer’s instructions. Cells transfected with the GHSR-1a-specific siRNA and cells transfected with the nonspecific siRNA sequence (negative control) were set as the GHSR-1a-siRNA group and NC-siRNA group, respectively. Untransfected cells were used as the control group. The expression of GHSR-1a was examined 24 h after transfection by using reverse transcription polymerase chain reaction (RT-PCR).

### RT–PCR

To determine the mRNA level of GHSR-1a, total RNA was extracted from HRMECs using an RNA extraction kit (Thermo Fisher Scientific, USA) according to the manufacturer’s protocol. To synthesize cDNA, a total of 2 μg of mRNA was subjected to RT-PCR using HiScript^®^ II Q RT SuperMix (Vazyme, Nanjing, China). Quantitative real-time PCR was performed using SYBR Green Master Mix (Vazyme, Nanjing, China) in a PCR system (QuantStudio 6.0, ABI, USA). The primers (Tsingke Biotechnology Co., Ltd., Beijing, China) used for target mRNA detection were as follows: GHSR (forward 5′-CTACAGTCTCATCGGCAGGA-3′, reverse 5′-GAGAGAAGGGAGAAGGCACA-3′); GAPDH (forward 5′-TCAAGAAGGTGGTGAAGCAGG-3′, reverse 5′-TCAAAGGTGGAGGAGTGGGT-3′). The results are presented as values normalized to glyceraldehyde-3-phosphate dehydrogenase (GAPDH) expression using the 2^−ΔΔCt^ method.

### Cell migration

Cell migration was analyzed using Transwell chambers (Corning, USA). HRMECs from each group were digested with 0.25% trypsin, and a single-cell suspension was prepared with serum-free M199 medium at a density of 3 × 10^5^ cells/mL. Two hundred microlitres of the cell suspension was added to the upper compartment of the Transwell chamber, and M199 medium containing 10% FBS was added to the lower compartment. After 24 h of incubation, the Transwell chamber was removed. The cells were carefully cleaned with PBS, fixed with 70% iced ethanol solution for 1 h and stained with 0.5% crystal violet for 20 min. Nonadherent cells were removed by wiping with a swab, and the chamber was observed under a microscope. Five high magnification fields were randomly selected for counting the cells that crossed the membrane.

### Tube formation

Each well of 24-well plates was coated with 200 μL of Matrigel (Corning, USA) and placed at 37 °C for 60 min. Treated cells were digested with 0.25% trypsin, and single-cell suspensions were prepared with serum-free M199 medium. After counting, the cells were seeded evenly into a 24-well plate precoated with Matrigel at 1 × 10^5^ cells/well and cultured at 37 °C for 12 h. Images were acquired (IX51 Olympus, Japan), and the tube-like structures were analyzed using ImageJ (NIH, USA) at 100 × magnification.

### Western blotting

Total protein was extracted from cells with RIPA lysis buffer (Beyotime, China) after incubation for 48 h. After quantification of the protein concentration with a BCA protein assay kit (Beyotime, China), proteins in the samples (40 μg) were separated by SDS-PAGE and transferred to polyvinylidene fluoride (PVDF) membranes. Then, the membranes were blocked with 5% fat-free milk and incubated with diluted primary rabbit polyclonal antibodies, including anti-GHSR-1a (1:1000, Affinity, USA), anti-PERK (1:1000, Affinity, USA), anti-p-PERK (1:2000, Affinity, USA), anti-AFT4 (1:1000, Affinity, USA), anti-CHOP (1:1000, Affinity, USA), and anti-GAPDH (1:1000, Hangzhou Xianzhi Biology Co., Ltd., China) antibodies, overnight at 4 °C. Then, the membranes were incubated with horseradish peroxidase-conjugated goat anti-rabbit IgG (1:10,000, Boster, China) as the secondary antibody for 2 h at room temperature. The bands were visualized by ECL and quantified using Bandscan software 5.0 (Glyko Inc., CA, USA).

### Statistical analysis

Quantitative data are presented as the mean ± standard deviation (SD) of three independent experiments. SPSS 19.0 (IBM, USA) was used for conducting a pairwise *t* test or one-way analysis of variance (ANOVA) followed by a LSD post hoc test. Differences between or among the groups were statistically significant when *P* < 0.05.

## Results

### The expression of GHSR-1a in HRMECs

**Fig. 1:**
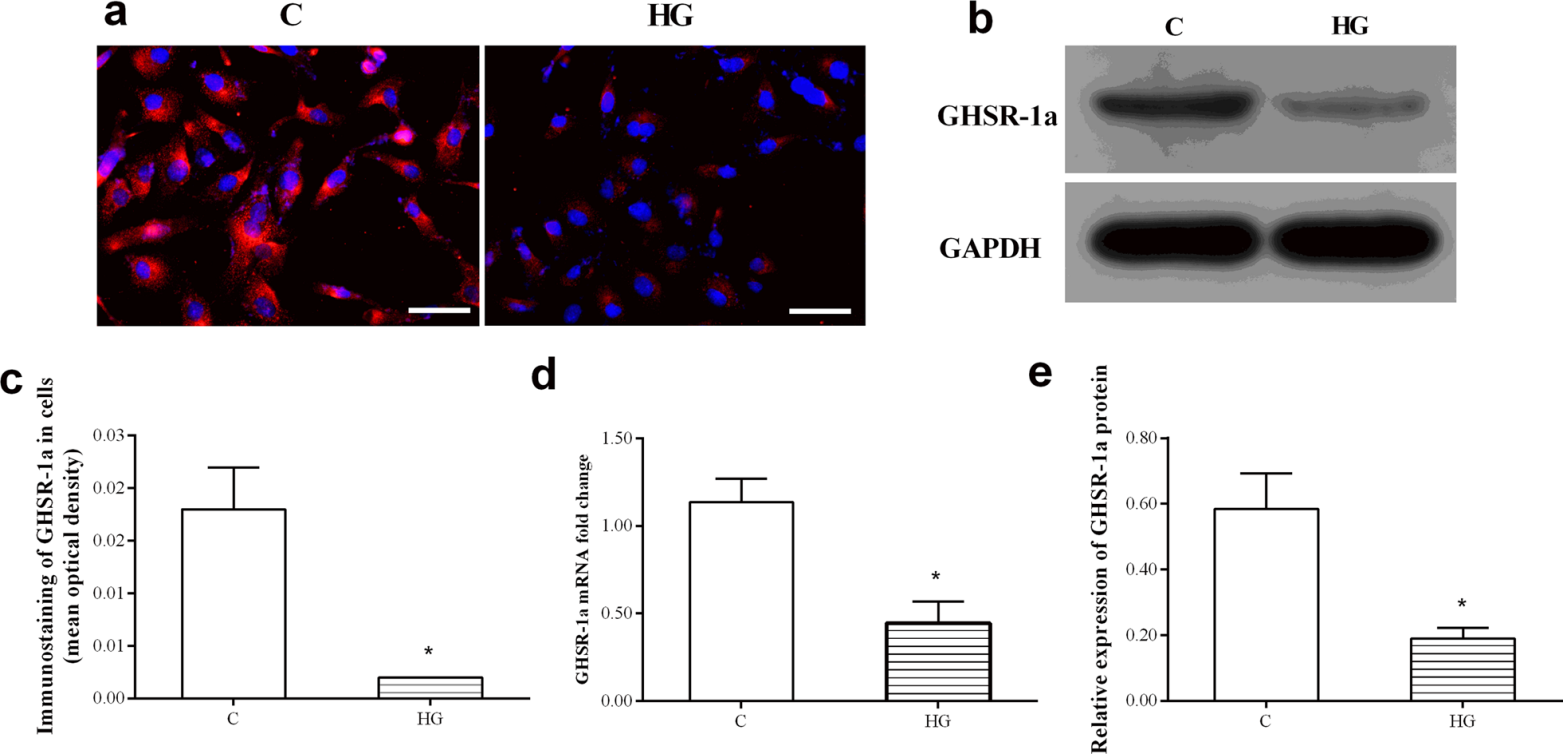
Representative immunofluorescence images (**a**, bar = 50 μm) and Western blotting (**b**) of the expression of GHSR-1a in human retinal microvascular endothelial cells (HRMECs) in different groups treated for 48 h. Statistical comparison of optical density (**c**), mRNA fold change (**d**), and relative protein expression of GHSR-1a (**e**) (n = 3, *P < 0.05 vs. C group). C, control; HG, high glucose

To study the effects of ghrelin, we first observed whether its receptor GHSR-1a is expressed in HRMECs. Real-time PCR, immunostaining and Western blotting showed that GHSR-1a was present in HRMECs at both the mRNA and protein levels. In addition, high glucose markedly inhibited the expression of GHSR-1a when compared with that in the control group (Fig. 1).

### Screening of ghrelin and siGHSR-1a

**Fig. 2:**
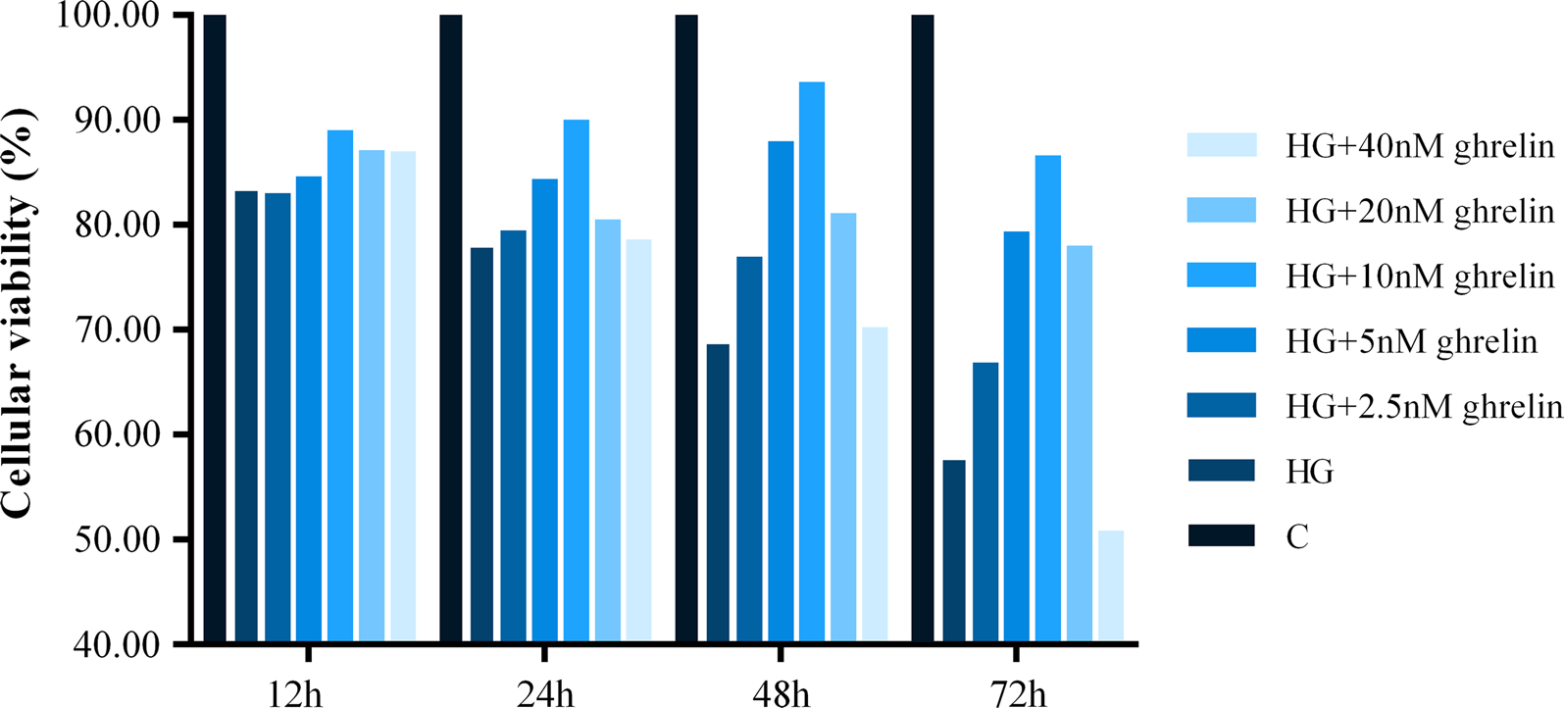
Cellular viability of human retinal microvascular endothelial cells (HRMECs) at 12 h, 24 h, 48 h and 72 h of treatment by CCK-8 assay in different groups. C, control; HG, high glucose

**Fig. 3:**
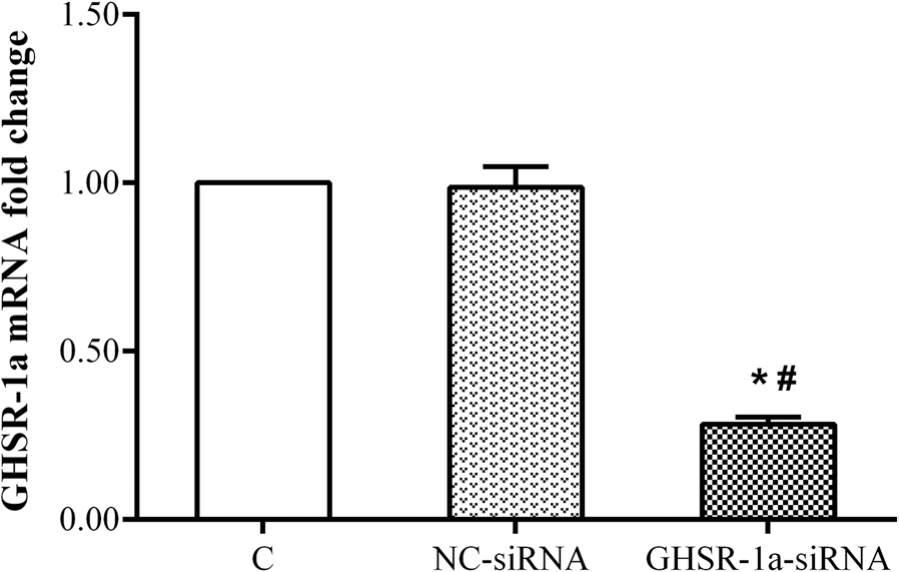
The mRNA expression level of GHSR-1a in human retinal microvascular endothelial cells (HRMECs) using RT-PCR in different groups (n = 3,*P < 0.05 vs. C group; ^#^*P* < 0.05 vs. NC-siRNA group). C, cells without transfection; NC-siRNA, cells transfected with nonspecific sequence (negative control); GHSR-1a-siRNA, cells transfected with GHSR-1a-specific siRNA

To determine the optimal ghrelin concentration and application time for treating HRMECs, concentrations of 2.5 nM, 5 nM, 10 nM, 20 nM, and 40 nM and times of 12 h, 24 h 48 h and 72 h were evaluated. Based on the results of the CCK-8 assay (Fig. 2), treatment with 10 nM ghrelin for 48 h was used for all subsequent studies. Then, to prove that ghrelin functions through its receptor GHSR-1a, siRNA transfection was used, and successful silencing of GHSR-1a was achieved (Fig. 3).

### The effects of ghrelin on the viability, migration, and tube formation of HRMECs under high-glucose conditions

**Fig. 4:**
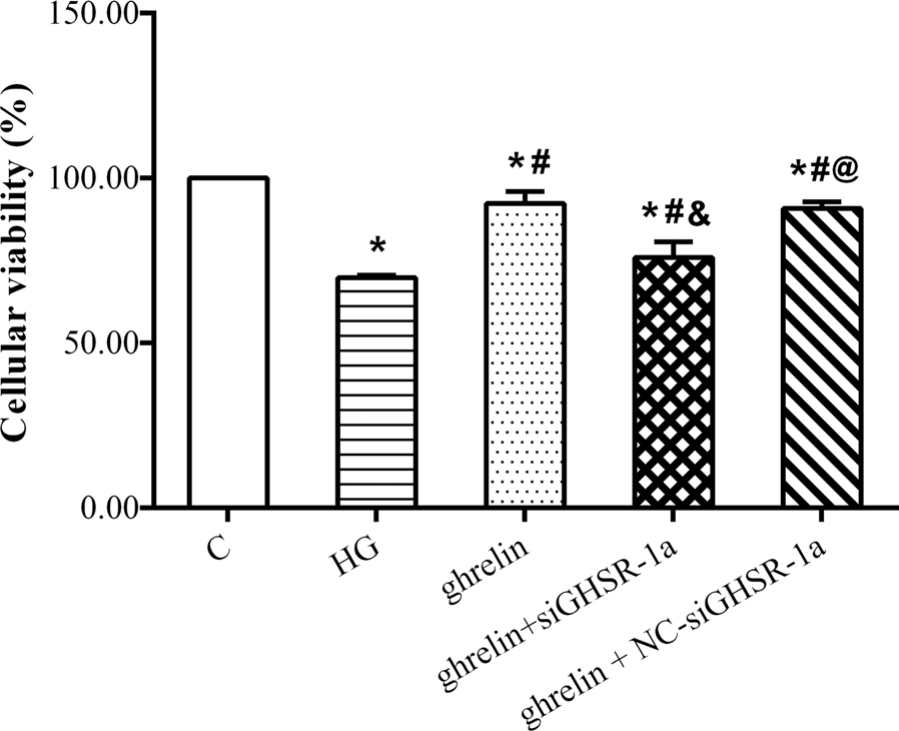
Cellular viability of human retinal microvascular endothelial cells (HRMECs) at 48 h of treatment by CCK-8 assay in different groups (n = 3, *P < 0.05 vs. C group; ^#^*P* < 0.05 vs. HG group; ^&^*P* < 0.05 vs. ghrelin group; ^@^*P* < 0.05 vs. ghrelin + siGHSR-1a group). C, control; HG, high glucose; siGHSR-1a, cells transfected with GHSR-1a-specific siRNA; NC-siGHSR-1a, cells transfected with nonspecific sequence (negative control) of siGHSR-1a

**Fig. 5:**
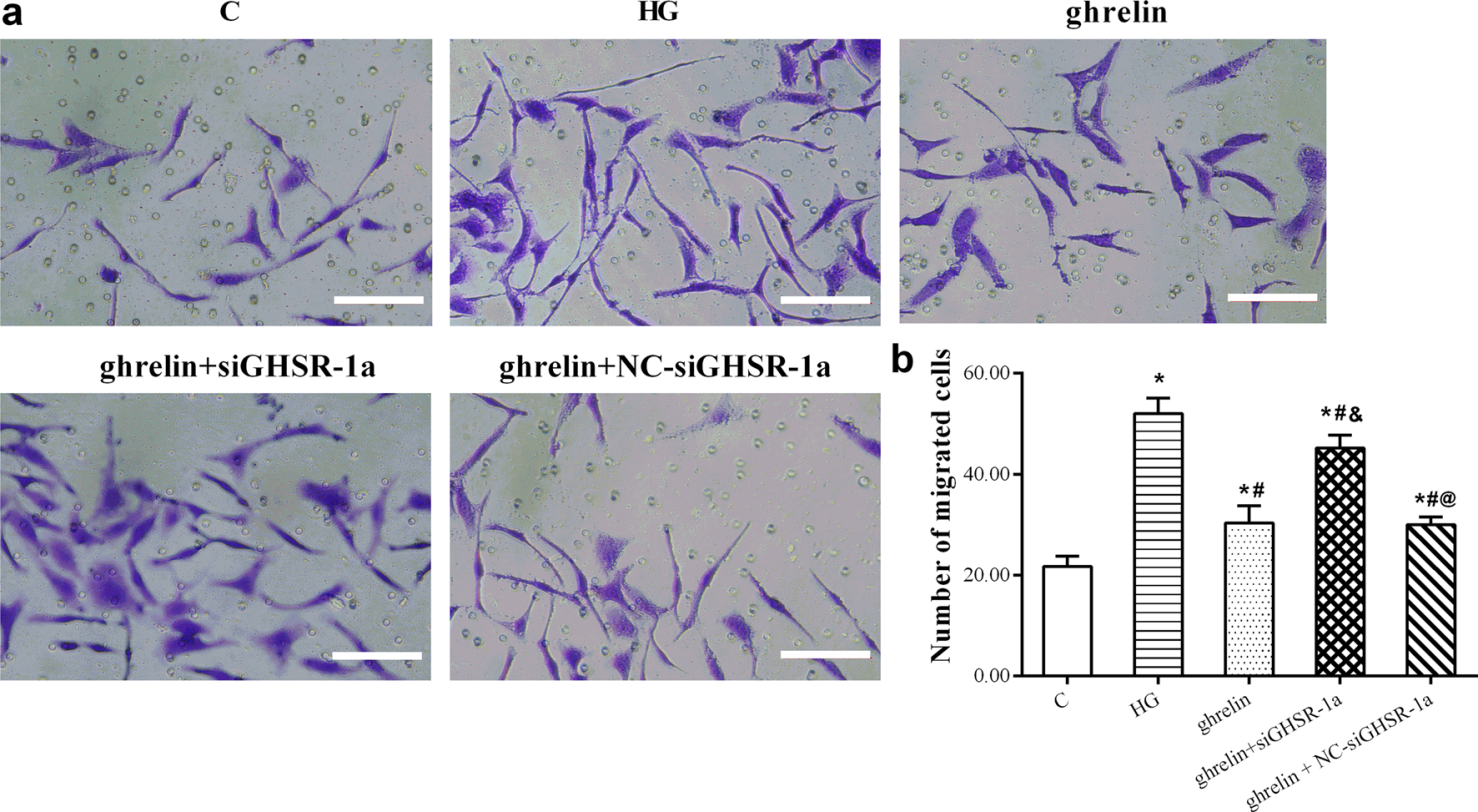
Representative images of migration of human retinal microvascular endothelial cells (HRMECs) at 48 h of treatment by Transwell assay in different groups (**a**, bar = 100 μm), and the statistical analysis for the number of migrated cells (**b**) (n = 3, *P < 0.05 vs. C group; ^#^*P* < 0.05 vs. HG group; ^&^*P* < 0.05 vs. ghrelin group). C, control; HG, high glucose; tunicamycin, an inducer of endoplasmic reticulum stress

**Fig. 6:**
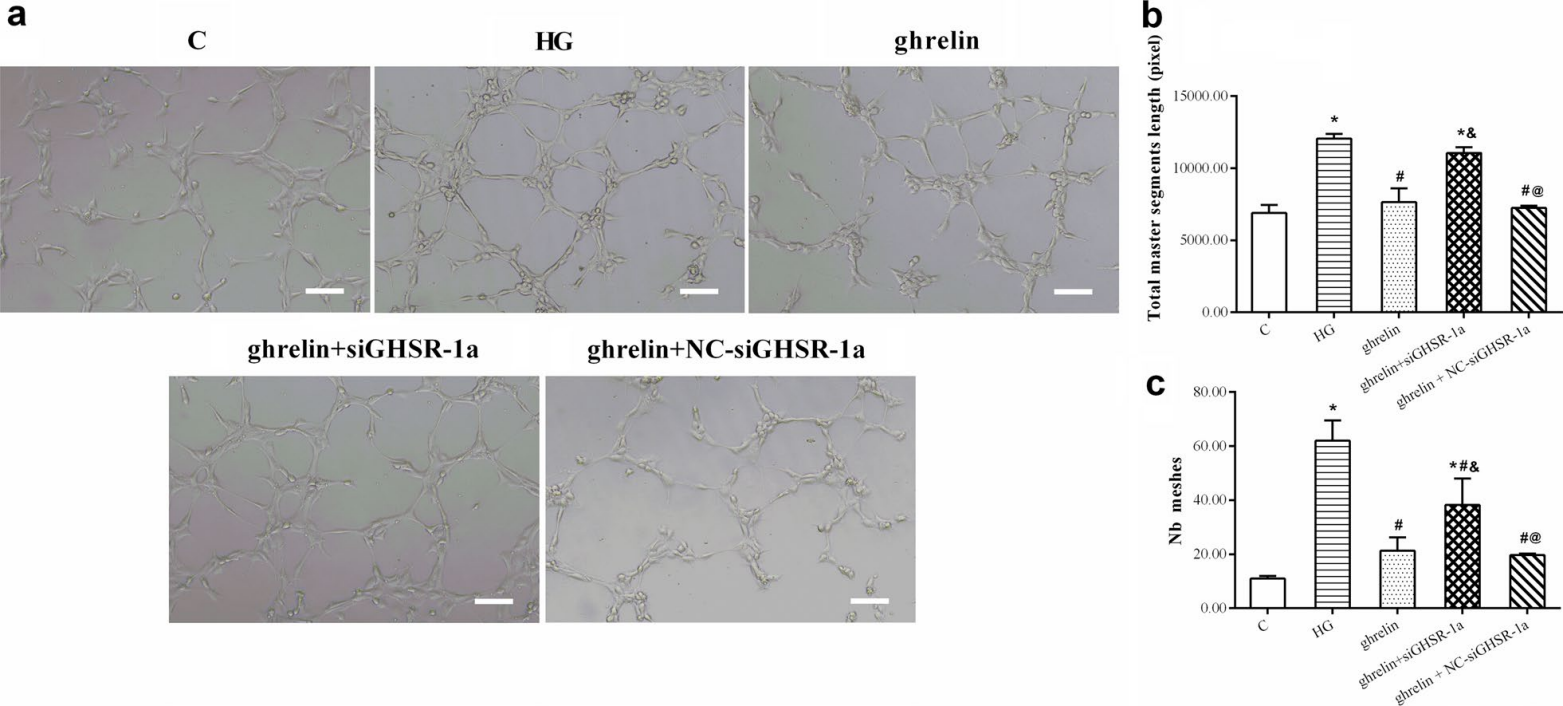
Representative images of tube formation (**a**, bar = 100 μm) of human retinal microvascular endothelial cells (HRMECs) at 48 h of treatment by Matrigel assay in different groups, and the statistical analysis for the total master segments length (**b**) and Nb meshes (**c**) (n = 3, *P < 0.05 vs. C group; ^#^*P* < 0.05 vs. HG group; ^&^*P* < 0.05 vs. ghrelin group; ^@^*P* < 0.05 vs. ghrelin + siGHSR-1a group). C, control; HG, high glucose; siGHSR-1a, cells transfected with GHSR-1a-specific siRNA; NC-siGHSR-1a, cells transfected with nonspecific sequence (negative control) of siGHSR-1a

Under high-glucose conditions, the viability of HRMECs decreased significantly, and their migration and tube formation abilities increased. When ghrelin was added, viability was enhanced, while migration and tube formation were inhibited. When siGHSR-1a was added, these changes were reversed when compared with those in cells without ghrelin treatment or with combined ghrelin and NC-siGHSR-1a treatment (Figs. 4, 5, 6). These results suggest that Ghrelin protects the survival of HRMECs and inhibits high glucose-induced angiogenesis through GHSR-1a.

### The effects of ghrelin on ER stress in HRMECs under high-glucose conditions

Under high-glucose conditions, the protein levels of p-PERK, ATF4 and CHOP in HRMECs were increased, indicating that ER stress was significantly activated. However, when ghrelin was added, the levels of these proteins were decreased by almost half.

**Fig. 7:**
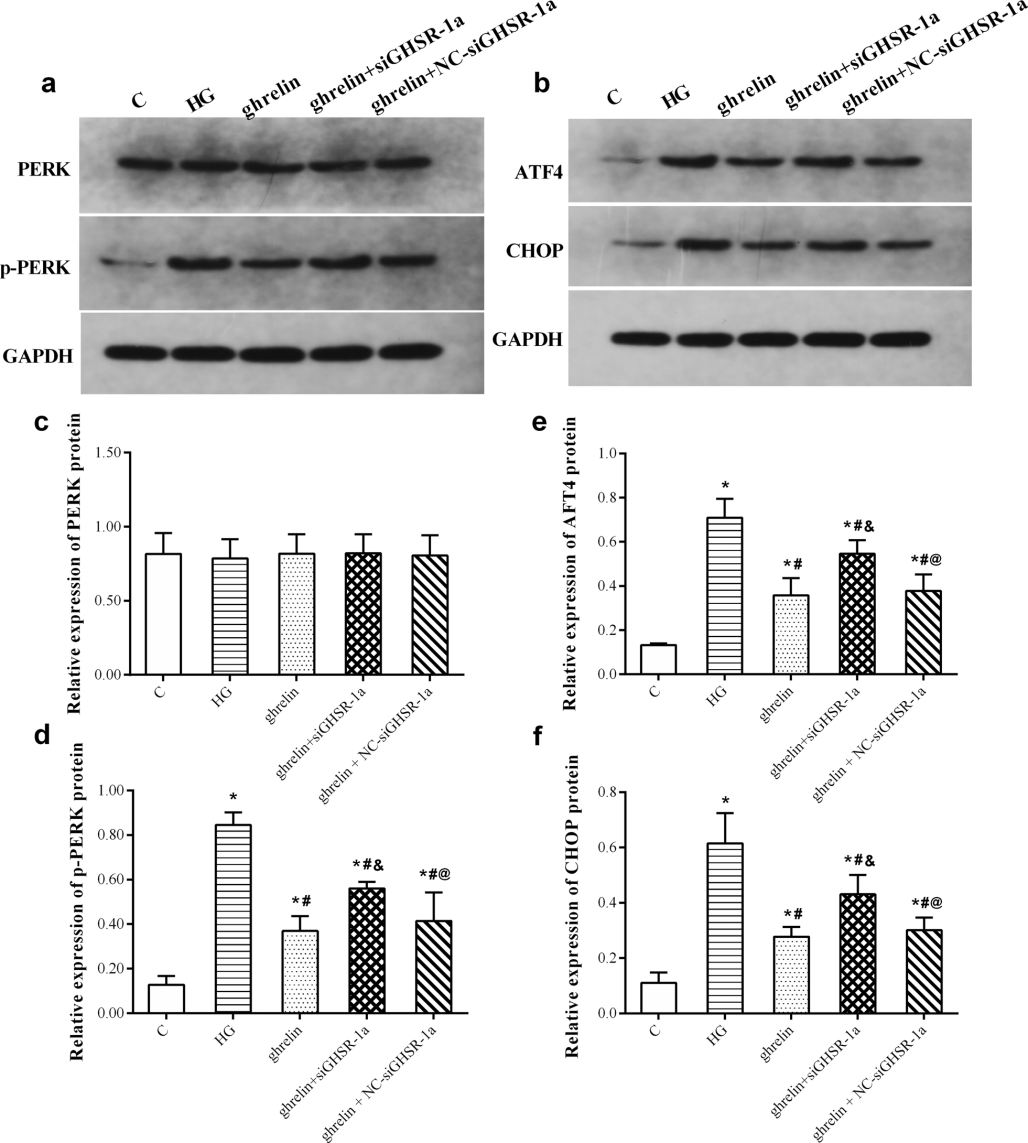
Representative Western blotting images of PERK, p-PERK, ATF4, and CHOP in human retinal microvascular endothelial cells (HRMECs) at 48 h of treatment in different groups (**a**, **b**), and the relative expression of PERK (**c**), p-PERK (**d**), ATF4 (**e**), and CHOP (**f**) after normalization by GAPDH (n = 3, *P < 0.05 vs. C group; ^#^*P* < 0.05 vs. HG group; ^&^*P* < 0.05 vs. ghrelin group; ^@^*P* < 0.05 vs. ghrelin + siGHSR-1a group). C, control; HG, high glucose; siGHSR-1a, cells transfected with GHSR-1a-specific siRNA; NC-siGHSR-1a, cells transfected with nonspecific sequence (negative control) of siGHSR-1a; GAPDH, glyceraldehyde-3-phosphate dehydrogenase

In addition, this effect of ghrelin was markedly suppressed by knockdown of its receptor GHSR-1a, as shown by the higher levels of these proteins (Fig. 7).

### The effects of ghrelin on high glucose-induced angiogenesis of HRMECs and ER stress

**Fig. 8:**
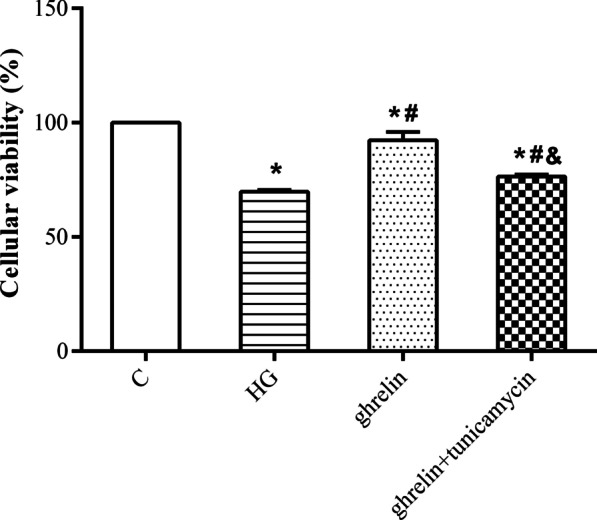
Cellular viability of human retinal microvascular endothelial cells (HRMECs) at 48 h of treatment by CCK-8 assay in different groups (n = 3, *P < 0.05 vs. C group; ^#^*P* < 0.05 vs. HG group; ^&^*P* < 0.05 vs. ghrelin group). C, control; HG, high glucose; tunicamycin, an inducer of endoplasmic reticulum stress

**Fig. 9:**
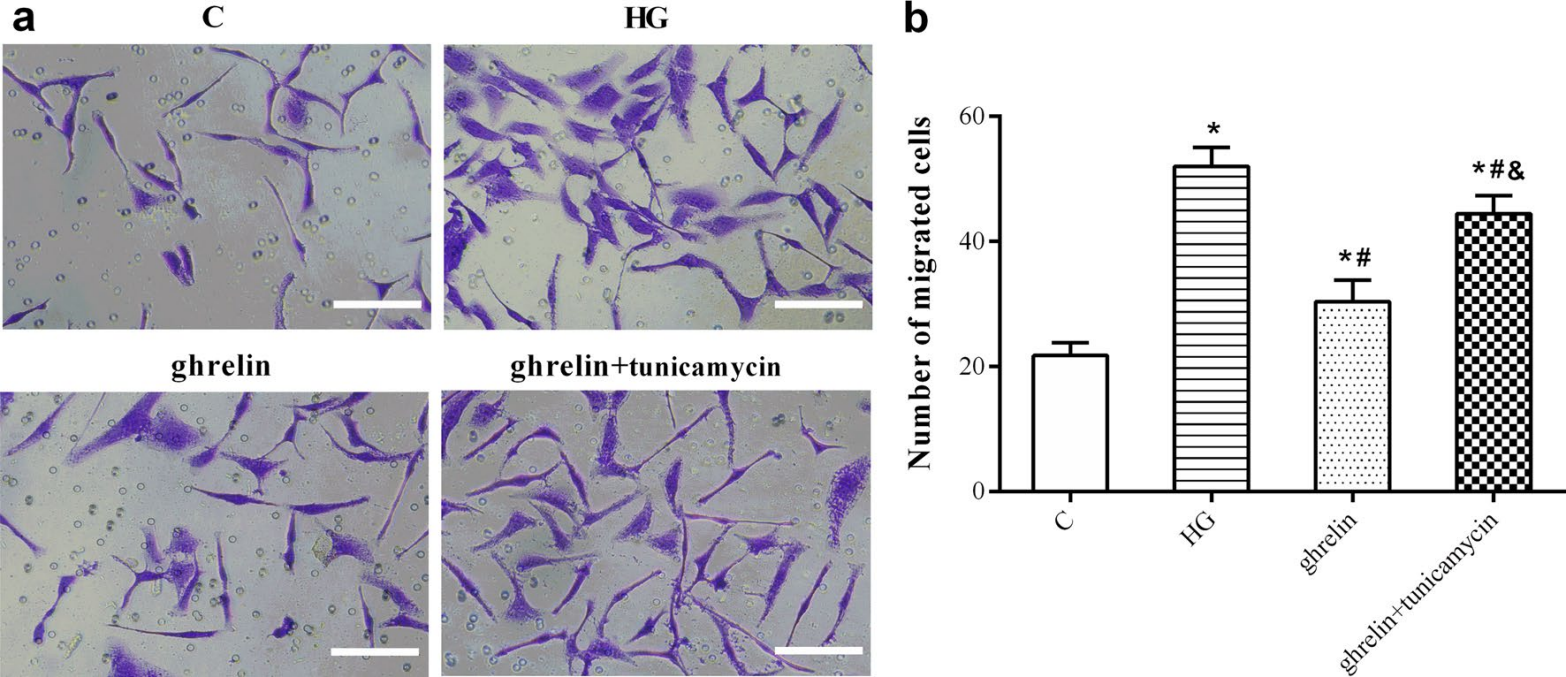
Representative images of migration (**a**, bar = 100 μm) of human retinal microvascular endothelial cells (HRMECs) at 48 h of treatment by Transwell assay in different groups, and the statistical analysis for the number of migrated cells (**b**) (n = 3, *P < 0.05 vs. C group; ^#^*P* < 0.05 vs. HG group; ^&^*P* < 0.05 vs. ghrelin group; ^@^*P* < 0.05 vs. ghrelin + siGHSR-1a group). C, control; HG, high glucose; siGHSR-1a, cells transfected with GHSR-1a-specific siRNA; NC-siGHSR-1a, cells transfected with nonspecific sequence (negative control) of siGHSR-1a

**Fig. 10:**
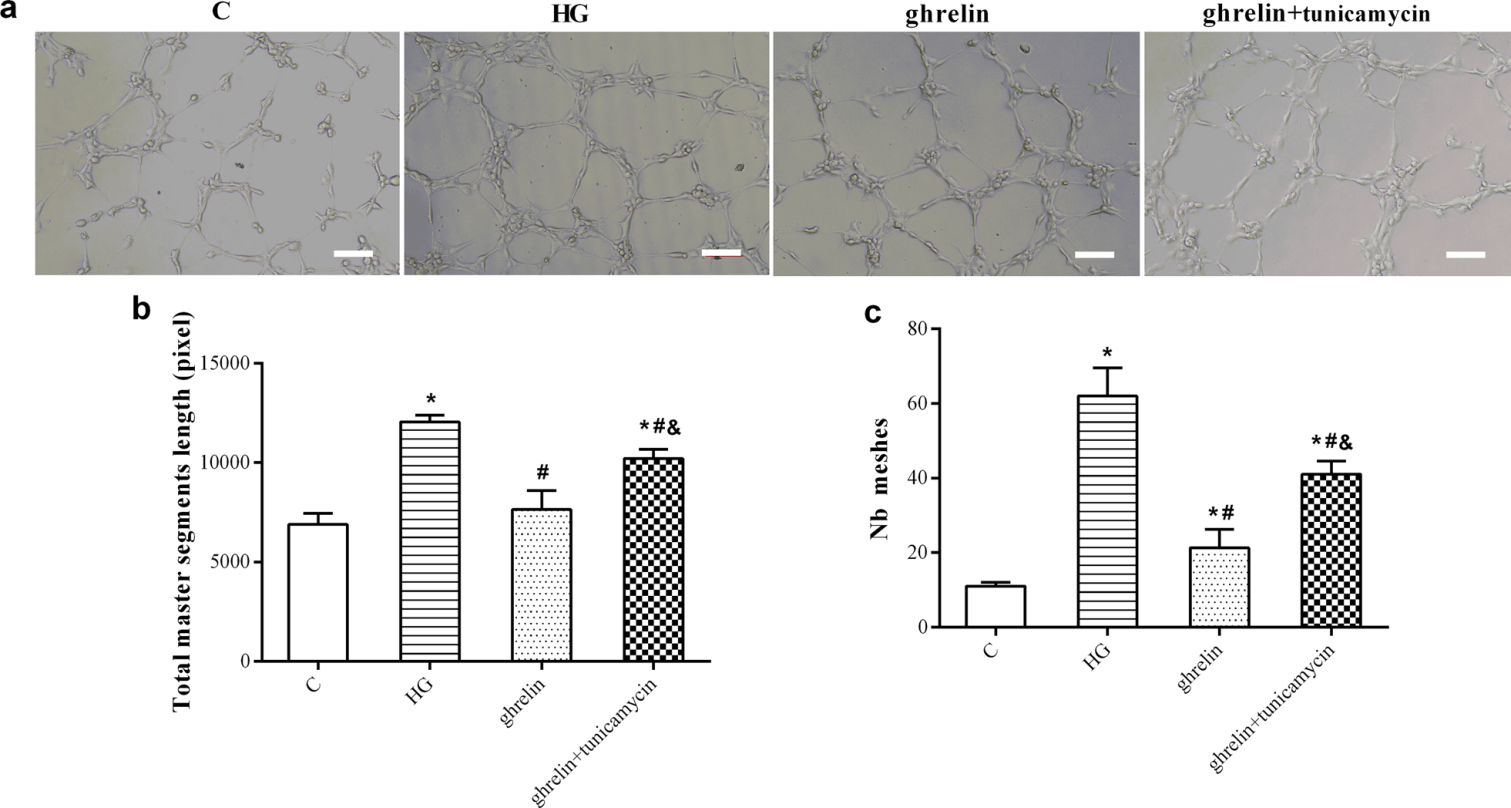
Representative images of tube formation of human retinal microvascular endothelial cells (HRMECs) at 48 h of treatment by Matrigel assay in different groups (**a**, bar = 100 μm), and the statistical analysis of the total master segments length (**b**) and Nb meshes (**c**) (n = 3, *P < 0.05 vs. C group; ^#^*P* < 0.05 vs. HG group; ^&^*P* < 0.05 vs. ghrelin group). C, control; HG, high glucose; tunicamycin, an inducer of endoplasmic reticulum stress

To investigate the hypothesis that ER stress is a key target for the function of ghrelin as an inhibitor of high glucose-induced angiogenesis, we used tunicamycin, an inducer of ER stress. As expected, the effects of ghrelin on cell viability, migration and tube formation under high-glucose conditions were weakened by the addition of tunicamycin (Figs. 8, 9, 10). These results indicated that ghrelin could protect HRMECs and inhibit the process of angiogenesis by inhibiting high glucose-induced ER stress.

## Discussion

In 1999, Kojima et al. [21] was the first to identify ghrelin, a new brain-gut peptide hormone, in the endocrine cells of rat and human gastric mucosa and the arcuate nucleus of the hypothalamus and determined that it regulates growth hormone release. As a relatively novel multifaceted hormone, ghrelin was found in subsequent studies to exert a plethora of physiological effects. It is reported that ghrelin can increase food intake, affect sleep rhythm, regulate pancreatic endocrine function, regulate glucose metabolism and reduce fat utilization to finally achieve a positive energy balance in the body [10]. Ghrelin consists of 28 amino acid residues and has an approximate molecular weight of 3314 Da. As an endocrine or paracrine factor, ghrelin acts on GHSR, which is widely distributed in the central nervous system and peripheral tissues [22]. GHSR is divided into types 1a and 1b, and GHSR-1a is currently the only known receptor that can bind to ghrelin [23]. To investigate the effect of ghrelin on HRMEC angiogenesis under high-glucose conditions, the expression of GHSR-1a in the cells was first evaluated in this study. The RT-PCR, Western blotting and immunofluorescence assay results showed that GHSR-1a is expressed in HRMECs, similar to the findings in rat retinal vascular endothelial cells [15]. Previous studies have found that ghrelin and GHSR-1a are also expressed in the pigment epithelium of the ciliary body, retinal pigment epithelium (RPE) and iris in the human eye [24], and their expression can also be detected in the aqueous humor [25], suggesting that ghrelin plays a regulatory role in the physiological function of the eye and is closely related to the occurrence of eye diseases. In this study, GHSR-1a was detected in HRMECs, and the expression of GHSR-1a in HRMECs under high-glucose conditions was significantly lower compared with the control group, indicating that the downregulation of ghrelin-GHSR-1a signaling may be related to retinal vascular injury in diabetes.

To observe the protective effect of ghrelin on HRMECs under high-glucose conditions, the effects of different concentrations of ghrelin on cell viability were evaluated. The results showed that a concentration of 10 nM and an application time of 48 h were the most appropriate conditions for ghrelin treatment. In subsequent studies, GHSR-1a siRNA was used to block the effect of ghrelin. The results showed that the viability of HRMECs in the high-glucose group was significantly reduced, consistent with our previous results [26]. The viability of cells treated with exogenous ghrelin was significantly greater than that of cells in the high-glucose group. However, after blockade of GHSR-1a, the promoting effect of ghrelin on cell viability was significantly attenuated. Angiogenesis reflects the early process of capillary formation and is an indicator of endothelial cell (EC) function in vitro. The most commonly used assays to evaluate in vitro angiogenesis are migration, proliferation, and tube formation assays of ECs in response to inhibitory or stimulatory compounds [27]. In this study, the migration and tube formation of HRMECs were investigated, and high-glucose treatment was found to significantly enhance the two steps of retinal angiogenesis, consistent with previous reports [28, 29]. When ghrelin was used in combination with high-glucose treatment, the migration and tube formation abilities of HRMECs were weakened, and siGHSR-1a restored the promoting effects of high glucose on these two steps of angiogenesis. Similar to our study, some studies have shown that ghrelin can inhibit vascular endothelial cell angiogenesis [30, 31]; however, other studies have found opposite results suggesting that ghrelin can boost angiogenesis [32, 33]. Taking these findings together, we hypothesized that ghrelin has a protective effect on vascular endothelial cells [34] and maintains cellular homeostasis under pathological conditions by regulating—either promoting or inhibiting—angiogenesis [15], for cells to better adapt to the environment.

The ER is an important organelle responsible for calcium storage, calcium homeostasis and protein folding and processing. When the function of the ER is impaired by external stimulation, disruption of intracellular calcium homeostasis and accumulation of misfolded or unfolded proteins can induce the ER stress response. At the early stage of ER stress, cells initiate the unfolded protein response (UPR) to protect cells from ER stress and maintain normal physiological functions. When stress is sustained, the homeostasis of the intracellular environment is destroyed, and apoptosis is induced via activation of the PERK, IRE1, and ATF6 pathways [35]. Many studies have shown that ER stress is related to the pathogenesis of DR, which is involved in the death of both retinal neurons and vascular cells in diabetic eyes [19, 36]. Therefore, reducing or blocking ER stress may be a potential therapeutic approach for preventing the onset and progression of DR. Here, we explored whether the effects of ghrelin on HRMECs are related to ER stress under high-glucose conditions. Ghrelin greatly decreased the high glucose-induced overexpression of ER stress marker proteins in the PERK pathway, including PERK, ATF4 and CHOP [37], in HRMECs. When tunicamycin, an inducer of ER stress, was added, the promoting effect of ghrelin on cell viability was attenuated. The inhibitory effects of ghrelin on the migration and tube formation of HRMECs were also attenuated by tunicamycin. These results suggested that the effects of ghrelin on HRMECs under high-glucose conditions were mediated through inhibition of ER stress.

However, it is still unknown whether ghrelin can inhibit the development of DR as it does in other diabetic microvascular complications. Therefore, future studies are necessary to elucidate the protective role of ghrelin in DR and determine the molecular mechanisms via in vivo experiments. Currently, there have been no reports on the toxicity of ghrelin. In general, ocular drug delivery is an important issue; thus, the stability of ghrelin should also be considered. We performed studies only in vitro; therefore, to translate these promising findings into clinical practice, an innovative ocular delivery nanosystem for ghrelin could be useful to develop in the future [38].

## Conclusions

The results of this study preliminarily confirmed the protective effect of ghrelin on cell viability as well as its inhibitory effect on retinal vascular endothelial cell angiogenesis under high-glucose conditions. Inhibition of ER stress induced by high glucose may be one of the mechanisms by which ghrelin mediates.

## Data Availability

The data sets used and/or analyzed during the current study are available from the corresponding author on reasonable request.
